# Changes in the Proteome of *Medicago sativa* Leaves in Response to Long-Term Cadmium Exposure Using a Cell-Wall Targeted Approach

**DOI:** 10.3390/ijms19092498

**Published:** 2018-08-24

**Authors:** Annelie Gutsch, Salha Zouaghi, Jenny Renaut, Ann Cuypers, Jean-Francois Hausman, Kjell Sergeant

**Affiliations:** 1 Environmental Research and Innovation, Luxembourg Institute of Science and Technology, 5, avenue des Hauts-Fourneaux, Esch-sur-Alzette, 4362 Luxembourg, Luxembourg; annelie.gutsch@list.lu (A.G.); salhazouaghi@gmail.com (S.Z.); jenny.renaut@list.lu (J.R.); jean-francois.hausman@list.lu (J.-F.H.); 2 Agoralaan building D, Hasselt University, Campus Diepenbeek, Centre for Environmental Science, 3590 Diepenbeek, Belgium; ann.cuypers@uhasselt.be

**Keywords:** *Medicago sativa*, leaf cell wall proteome, cadmium, quantitative proteomics, 2D DIGE

## Abstract

Accumulation of cadmium (Cd) shows a serious problem for the environment and poses a threat to plants. Plants employing various cellular and molecular mechanisms to limit Cd toxicity and alterations of the cell wall structure were observed upon Cd exposure. This study focuses on changes in the cell wall protein-enriched subproteome of alfalfa (*Medicago sativa*) leaves during long-term Cd exposure. Plants grew on Cd-contaminated soil (10 mg/kg dry weight (DW)) for an entire season. A targeted approach was used to sequentially extract cell wall protein-enriched fractions from the leaves and quantitative analyses were conducted with two-dimensional difference gel electrophoresis (2D DIGE) followed by protein identification with matrix-assisted laser desorption/ionization (MALDI) time-of-flight/time of flight (TOF/TOF) mass spectrometry. In 212 spots that showed a significant change in intensity upon Cd exposure a single protein was identified. Of these, 163 proteins are predicted to be secreted and involved in various physiological processes. Proteins of other subcellular localization were mainly chloroplastic and decreased in response to Cd, which confirms the Cd-induced disturbance of the photosynthesis. The observed changes indicate an active defence response against a Cd-induced oxidative burst and a restructuring of the cell wall, which is, however, different to what is observed in *M. sativa* stems and will be discussed.

## 1. Introduction

Pollution of soil, water and air is one of the serious issues of recent decades. Amongst others, contamination with heavy metals is of great concern due to their stability in the ecosystem. Contaminated sites are inaccessible for humans in the context of urbanization, biomass- and food-production, which poses a major problem and exacerbates the already limited availability of soil. Cadmium (Cd) is one of the most common pollutants in the environment with a high degree of genotoxicity [1]. Plants exposed to Cd suffer from an impairment of physiological and biochemical processes. They show limited growth and chlorosis and Cd leads to oxidative stress by generating reactive oxygen species (ROS) [2]. Cadmium interferes with photosynthesis by reducing the chlorophyll content, depressing the photosynthetic rate and induces direct damage to photosynthetic enzymes in a concentration- and time-dependent manner. Thereby, it was shown that Cd interferes more profoundly with the activity of photosystem II than photosystem I [3,4]. Cadmium can displace calcium (Ca) in photosystem II, thus inhibiting the formation of a functional complex and preventing photoactivation [5].

The plant cell wall is a dynamic cell-surrounding structure, which provides mechanical support and rigidity. It consists of cellulose, hemicellulose, pectin, as well as phenolic compounds. Proteins responsible for intercellular communication and interaction between the cell and the environment are imbedded in the cell wall. Those proteins make about 10% of the cell wall mass and their tightly regulated enzymatic reactions can alter the cell wall structure and properties [6,7], not only during plant development, but also during plant defence responses to biotic and abiotic stress [8,9]. Pectin methylesterase (PME), a cell wall protein, de-esterifies the pectic polysaccharide homogalacturonan (HG) creating binding sites for Ca^2+^. Bound Ca mediates the bridging between two HG molecules to form a stable gel (egg-box structure) [10,11]. In the presence of Cd, PME showed an enhanced activity and the degree of low-methylesterified pectin in the cell wall increases concurrently with the deposition of Cd. By having the same charge, Cd^2+^ can bind pectin and displace Ca^2+^ as the cross-linking ion in the egg-box structure [12,13,14]. Additionally, Cd exposure has been shown to enhance lignification of the cell wall through an increased activity of cell wall-bound peroxidases, which causes cell wall stiffening and growth inhibition [15,16]. Such Cd-induced alterations of the cell wall structure indicate that the cell wall is part of the defence mechanisms set-up by the plant and that those structural changes limit further translocation of Cd, thus, keeping cytosolic Cd concentrations low.

The plant cell wall proteome has been studied in different species including dicots and monocots. To date, the *Arabidopsis thaliana* cell wall proteome is the most comprehensive [17]. Yet, the leaf apoplastic proteome including cell wall proteins remains much less studied [18] and information about cell wall proteins that change in abundance due to a treatment is underrepresented in the current scientific literature [19,20]. However, comparative cell wall proteome studies in leaves already provided information on how the cell wall proteome changes when exposed to various stresses [21,22]. To understand the mechanisms that take place in the cell wall during exposure to environmental constrains, it is important to unravel the cell wall proteome, its involvement in stress detection and response as well as its role in maintaining cell wall integrity.

*Medicago sativa*, commonly known as alfalfa, is an important forage legume and often used for research on cell wall development and stress adaptation [23,24]. Contrary to most research, in the present study *M. sativa* plants were exposed to realistic Cd concentrations for a long-term period, which makes the here-obtained results relevant for agricultural practices. Relative quantitative changes of the cell wall protein-enriched subproteome from leaves were investigated using 2D DIGE, which not only enables relative quantification but also visualizes different protein isoforms and modified proteins caused by Cd exposure [25]. A protocol for the enrichment of cell wall proteins was recently developed for *M. sativa* stems [26] and used in the current study on *M. sativa* leaves. The number of cytosolic contaminants in the different cell wall protein-enriched fractions remain low, which facilitates an accurate understanding of the leaf cell wall proteome. Although *M. sativa* proteins can be identified based on homology with *M. truncatula* proteins, as performed in a recent study [27], the combination of a search against the NCBI database and the *M. sativa* nucleotide database enlarges the number of identified proteins and the sequence coverage of the identified proteins, giving more comprehensive results. To our knowledge, this is the first study of the cell wall proteome of *M. sativa* leaves after long-term exposure to Cd.

## 2. Results

Cell wall protein-enriched fractions were obtained by subsequently using three different buffers of increasing ionic strength containing CaCl_2_, ethylene glycol-*bis*[β-aminoethyl ether]-*N*,*N*,*N*′,*N*′-tetraacetic acid (EGTA) or LiCl, to extract proteins with various wall-binding affinities. Using a targeted extraction protocol, the contamination with cytosolic proteins is low. However, several proteins involved in photosynthesis were identified and quantitative changes in these proteins are consistent throughout the fractions and replicates. As photosynthetic proteins are highly abundant in leaves and as Cd affects photosynthesis, they are included in the results and discussion.

**Figure 1:**
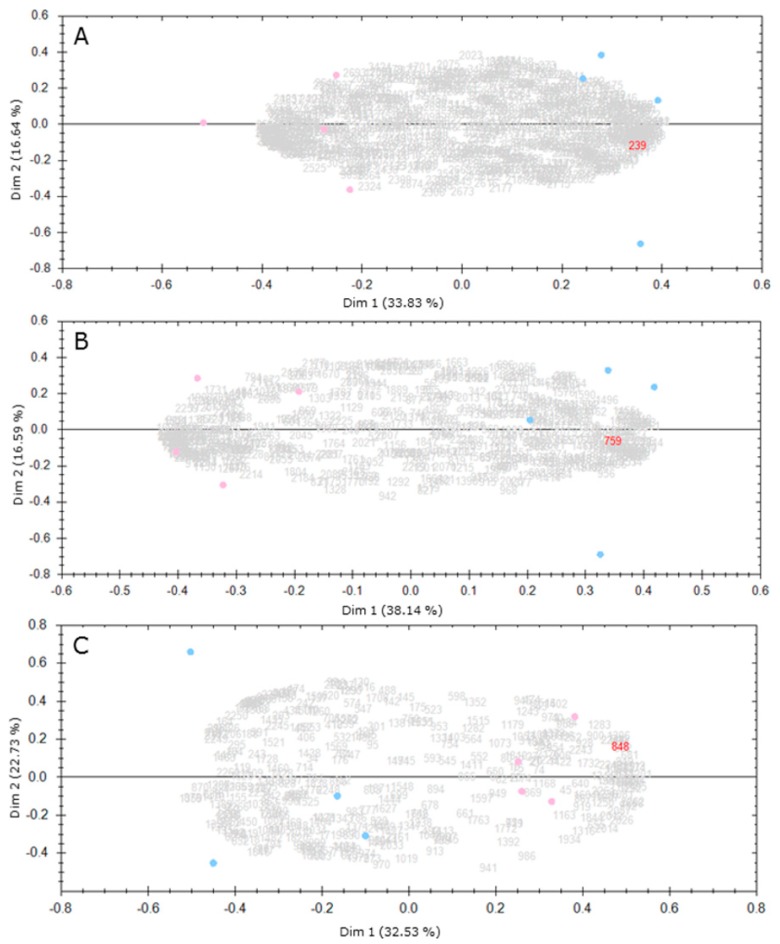
PCA analysis of the gel-based spot intensity data from the three cell wall protein-enriched fractions. (**A**) CaCl_2_; (**B**) EGTA; (**C**) LiCl. Statistical analysis was done with SameSpots software (TotalLab). Blue dots represent the four biological replicates of the control. Pink dots represent the four biological replicates of Cd-exposed samples. Grey and red numbers correspond to spot numbers considered for the statistical analysis.

A principle component analysis (PCA) on the gel-based spot intensity data analysed with the SameSpots software (TotalLab) revealed a clear distinction between control and Cd-exposed samples in the three cell wall protein-enriched fractions (Figure 1).

**Figure 2:**
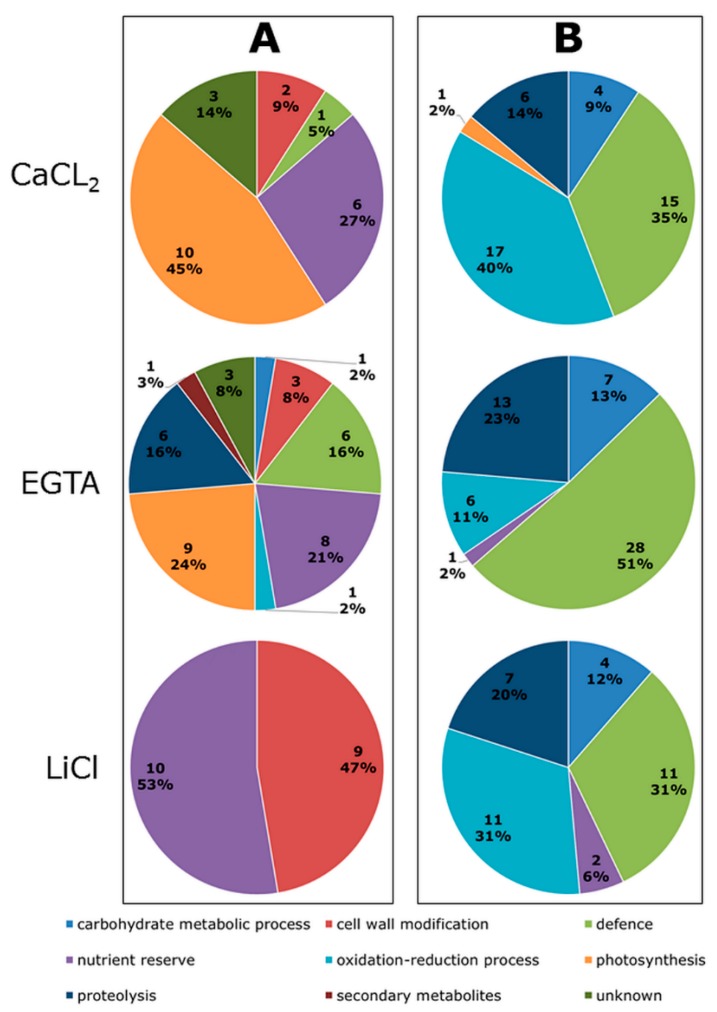
Functional classification of proteins present in cell wall protein-enriched fractions from *M. sativa* leaves after long-term exposure to Cd. *M. sativa* plants were exposed to Cd (10 mg/kg soil DW) for an entire season. Quantitative analysis based on four replicates were done with 2D DIGE comparing Cd-exposed samples to control samples and identified proteins clustered according to their predicted function using Blast2Go. (**A**) Functional classes of lower abundant proteins; and (**B**) functional classes of higher abundant proteins.

After manual and statistical evaluation of all detected spots, 306 spots showed a significant abundance change in response to Cd exposure (fold-change ≥ 1.2, ANOVA *p*-value ≤ 0.05) and were picked for identification. All mass spectra (MS) and MS/MS data that resulted in the protein identifications using the MASCOT server are provided in Table S1. Out of the total number of significantly changed spots, in 212 a single protein was identified and those were considered for biological interpretation. Based on the prediction for the subcellular localization with TargetP, 163 (76.9%) of these proteins are predicted to be secreted. Thirty seven are targeted to the chloroplast and 12 do not have a predicted subcellular target site. These predictions are mostly coherent with those from DeepLoc (Table S2), whereby DeepLoc distinguished the different locations after a protein has entered the secretory pathway as the most important change compared to TargetP. However, ongoing research in our lab indicates that DeepLoc predictions are not always reliable and vacuolar proteins are designates as extracellular and vice versa. Therefore, we based ourselves on TargetP in the results and in the discussion. In the CaCl_2_ fraction, 65 spots gave a significant identification of a single protein, of which 22 are of lower abundance and 43 are of higher abundance in response to Cd-exposure. Most proteins were identified in the EGTA fraction. Here, 93 proteins were found to increase (55 proteins) or decrease (38 proteins) in abundance. In the LiCl fraction a total number of 54 proteins were identified, of which 19 decreased and 35 increased in abundance. All proteins were clustered according to their predicted biological function to gain better insight on their physiological role and how this can be related to the plant’s response during Cd exposure (Figure 2). A complete list of all spots containing a single protein identification and, therefore, considered for biological interpretation is provided in Table S2, including statistical values obtained by the SameSpots software (TotalLab, Newcastle upon Tyne, UK) and their biological function. This information is summarised in Table 1. In several spots the same nominal protein was identified, however, the observation that it was identified at a different pI and/or molecular weight indicates that this concerns proteoforms. An example of this is a C-terminal truncation of eight amino acids from chitinase (e.g., the spots EGTA 1225 and 1279) that is probably determining for its subcellular location. The observation of a semi-tryptic peptide, corresponding to a cleavage in the middle of the papain family cysteine protease active domain in the spot EGTA 1971 (Table S1) indicates that the degradation of this protein increases in Cd-exposed plants. In the spot LiCl 1250 the same protein was identified but this time with a semi-tryptic peptide corresponding to the start of the active domain, after removal of the N-terminal inhibitor domain. These observations are confirmed by the position of the spots on the gel (Figure S1). Only by using a protein-based method, gel-based or gel-free, such proof of post-translational events can be obtained.

A large part of the higher abundant proteins are involved in plant defence (Figure 2). Another class of proteins with higher abundance upon Cd exposure have a designated function in oxidation-reduction processes. This classification includes different peroxidase isoforms present in all three fractions, plus a plastocyanin-like domain protein identified in the EGTA fraction (Table 1). Likewise, some proteins involved in carbohydrate metabolic processes and proteolysis are found to be of higher abundance. A minor part has a nutrient reserve function (rhicadhesin receptor, auxin-binding protein ABP19a) or is involved in photosynthesis (photosystem I reaction centre subunit II) (Table 1).

The functions assigned to proteins with a decreasing abundance are more diverse in the CaCl_2_ and EGTA fraction (Figure 2). Proteins identified in the LiCl fraction were only classified in cell wall modification (47%) or nutrient reserve (53%) (Figure 2). In those 19 spots only three different proteins were identified, namely auxin-binding protein ABP19a, stem 28 kDa glycoprotein, and polygalacturonase non-catalytic protein (Table S2). Most proteins of lower abundance in the CaCl_2_ fraction are involved in photosynthesis (oxygen-evolving enhancer protein, ribulose-1,5-bisphosphate carboxylase/oxygenase (RuBisCO) small chain and PS II oxygen-evolving enhancer protein) (Table S2). Photosynthetic proteins are highly abundant in leaves. As expected, the highest percentage of proteins that are not predicted to be cell wall localized is found in the CaCl_2_ fraction. It must be noted that no spot corresponding to RuBisCO large chain, the main component of the leaf proteome, is present. This protein makes up more than 50% of the total leaf protein content and masking of lower abundant proteins by RubisCO large chain seems to be avoided using the here applied protocol for cell wall protein enrichment. Of the identified proteins, only the function of the plant/F18G18-200 protein, containing a DUF642 conserved domain, identified in the CaCl_2_ and EGTA fraction remains unknown. The protein shows a decreased abundance in response to Cd and a secretion signal peptide was detected.

**Figure 3:**
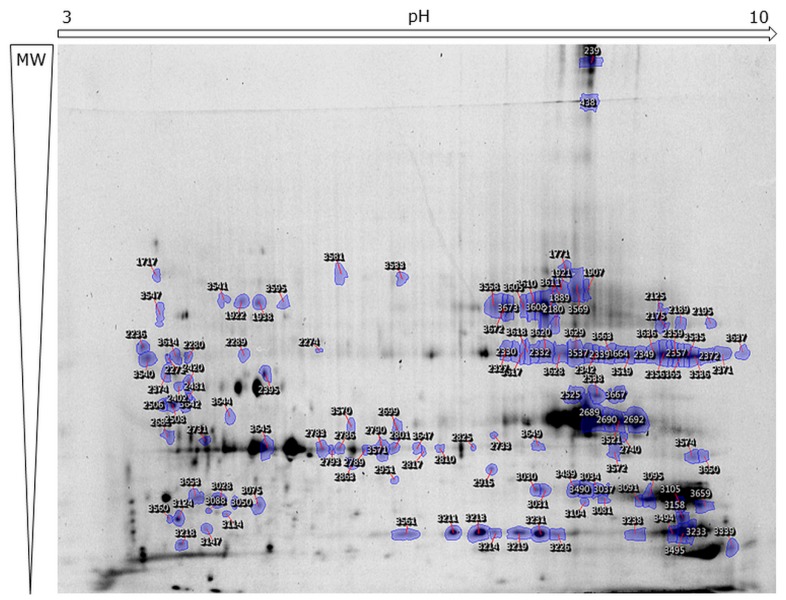
Illustration of 2D DIGE from the cell wall protein-enriched CaCl_2_ fraction from leaves of *M. sativa*. Extractions were performed in four replicates. Proteins were pre-labelled with different CyDye to enable relative quantitative protein analysis. Labelled samples were loaded onto Immobiline^TM^ DryStrip 3–10 NL, 24 cm (GE Healthcare) followed by migration on HPE^TM^ Large Gel NF-12.5% (Serva Electrophoresis GmbH). Indicated spots were selected for picking based on statistical parameters calculated by the SameSpots software (TotalLab). Images of 2D DIGE from EGTA and LiCl fractions are provided in Figure S1. MW = molecular weight.

Generally, if the same protein was identified in different spots, their changes in abundance were consistent with a comparable fold-change. However, spots containing auxin-binding protein ABP19a changed in different directions in the LiCl fraction (Table S2, spot 181 down and spot 1516 up) and were found to be less abundant in the CaCl_2_ fraction. Although the abundance of distinct isoforms of this protein is differently influenced by Cd exposure, the spectra of these proteins are identical and no hint was found to explain the observed dissimilarity. On the other side, of all spots containing CAP (cysteine-rich secretory protein, antigen 5) in the CaCl_2_ fraction (Table S2, spot 3105 and spots 3028, 3030, 3031, 3034, 3037, 3081, and 3095), only spot 3105 has a decreased abundance. All of these spots contain a CAP most homologous to *Medicaco truncatula* CAP gi: 357446161. Nonetheless, only in spot 3028 the N-terminal peptide, after removal of the signal peptide, is QDSQADYVNAHNEAR, corresponding to contig 56,806 of *M. sativa*. In the other spots QDSQADYVNAHNDAR is identified as an N-terminal peptide corresponding to the contigs 111,668 and 1437 (Table S1). When calculating the pI of the translated contigs, the position on the gel confirms that spot 3028 is more acidic than the others (Figure 3).

A number of spots in the EGTA fraction contain eukaryotic aspartyl protease family proteins. Some of these spots are of lower intensity after Cd-exposure (spot 956, 961, 962, 964, 978, and 983), while others are of higher intensity (904, 911, 917, 948, 955, 2239, and 2240, Table S2 and Figure S1). Based on the spectra and the matched peptide sequences (Table S1), it was found that aspartyl protease proteins with a decreasing abundance are predicted to be secreted, while those that are of increasing abundance have a chloroplast transit peptide. The distinction can also be observed on the gel images as the chloroplastic aspartyl proteases are acidic and thus cluster on the left side of the gel (Figure S1).

The secreted aspartyl proteases (EGTA fraction spot 956, 961, 962, 964, 978, and 983) can be divided into three groups according to their location on the gel: (1) 978, 983; (2) 956; and (3) 961, 962, 964 (Figure S1). The *M. sativa* contigs corresponding to those spots were blasted against the NCBI database and split into two different groups. Spots 978 and 983 (corresponding to contig 4015) show homology to the *M. truncatula* sequence XP_003594399.1, while the contigs corresponding to the remaining spots show the highest homology to the *M. truncatula* sequence XP_013459881.1. Those *M. truncatula* sequences only have a sequence identity of 37%. However, both proteins carry the same conserved domain (pepsin_retropepsin_like superfamily) and no functional differences could be found in the literature.

The larger groups of spots with the same functional annotation were dissected, for instance, in 29 spots different chitinase proteins were identified. These can be divided in five groups based on the most homologous *M. truncatula* protein. This grouping is in agreement with the calculated pI and molecular weight of the different spots. In the spots 1279, 2259, 1225, and 2249 of the EGTA fraction, a class I chitinase (gi:1800141) was identified. Surprisingly, the identified protein lacks the eight C-terminal amino acids as shown by the identification of the peptide at *m*/*z* 2683.19 (Table S1). Similar observations have been conducted before [28] and may relate to the fact that the protein is actually vacuolar and only removal of the C-terminal octopeptide allows the protein to be secreted [29]. Of the chitinase-containing spots, those spots that are matched to the gi:357443753 (spot 1399, 1416, 1419, and 2508) have the highest fold change (Table S2). No clear functional differences between the chitinases could be found during this analysis.

In addition to sequence variants, signal sequences and the cleavage of activation/inhibition sequences, other post-translational modifications were also observed during MS analysis. Among these, α-β didehydrophenylalanine, as a potentially structure-determining modification, was identified in the β-subunit of polygalacturonase [25].

## 3. Discussion

The current study shows the impact of long-term Cd exposure on the *M. sativa* leaf subproteome, enriched in cell wall proteins. Identified proteins were clustered according to their biological function using Blast2Go (Figure 2) and those assignments as well as the number of proteins showing an increased or decreased abundance upon Cd exposure are comparable with results from a similar study on *M. sativa* stems [30]. In both studies, results confirm that the enrichment obtained with the used protocol is better than that obtained with comparable protocols, but not 100% and several non-apoplastic proteins, mainly chloroplast targeted, were identified most prominent in the CaCl_2_ fraction (Figure 2) [30]. Those proteins will also be discussed as they are highly abundant in leaves and coherent, significant changes in abundance were observed between replicates and fractions in response to Cd exposure.

After planting, strong growth inhibition of Cd-exposed plants was observed at the end of the first growth cycle and coincided with the visual observation of leaf senescence. These phenotypical differences between Cd-exposed and control plants disappeared during the second growth cycle and no differences in appearance were observed at a later maturation state. Overall, long-term Cd exposure did not have any morphological impact on the leaves of *M. sativa*. Leaves from control and Cd-exposed plants had a rather heterogeneous appearance, due to which no leaf was representative for any of the conditions. Furthermore to attain the amount of leaf material needed, four to five grams for each replicate, all leaves from young to old were sampled. The visual observation of limited impact of long-term Cd exposure is supported by the fact that the average plant biomass taken from five replicates at the end of the experiment was not significantly different between Cd-exposed (120.32 ± 4.67 g) and control plants (110.65 ± 3.78 g) [30]. Although long-term experiments as presented here are rather scarce, the high sensitivity of initial growth stages followed by a low impact of the applied stress in adult plants was observed before for other plant species [15,31,32]. Obviously, plants do acquire a new steady-state when they are subjected to a constant severe but non-lethal stress [33].

### 3.1. Cd-Induced Degradation of Photosynthetic Proteins

Throughout all three cell wall protein-enriched fractions a decreasing abundance of photosynthetic proteins was revealed (Table 1). Among others, RuBisCO small chain was less abundant in Cd-exposed plants as well as subunits of photosystem I and II. The Cd-induced decreased abundance of photosynthetic proteins was recently reported in *M. sativa* stems after long-term Cd exposure when analysing cell wall protein-enriched fractions using 2D DIGE [30]. Likewise as in leaves, RuBisCO small chain, as well as a subunit of photosystem I, were less abundant in *M. sativa* stems when exposed to Cd. The fact that Cd is disrupting photosynthesis and that it causes oxidative stress in plants by ROS production is well known [2,34]. Reactive oxygen species are able to oxidise proteins, destining the oxidised protein for degradation, which goes together with an increasing protease activity [35,36]. Here, we observed that Cd induces a higher abundance of chloroplastic aspartyl protease isoforms in the EGTA fraction. The protein was identified before in leaf tissue of Cd-exposed *A. thaliana* [37] and its homology with cnd41-like proteins implies its function in senescence and nutrient recovery. Aspartyl proteases are implicated in degradation or processing of proteins and their occurrence during stress responses is established. They might have a crucial function in protein turn-over to prevent the accumulation of deactivated proteins, thereby increasing the pool of available amino acids, needed for the synthesis of defence-related proteins [38]. They are involved in RuBisCO degradation and degradation of chloroplastic proteins [39,40,41]. An increased abundance of this protease in *M. sativa* leaves correlates with the observed decreased abundance of photosynthetic proteins. Oxygen radicals, which appear during Cd exposure cause enhanced degradation of proteins involved in photosynthesis and impair the physiological processes in leaves. The increased abundance of a 70 kDa stromal heat shock protein (CaCl_2_ spot 2402) likewise indicates that there is an increased need for protein refolding in the chloroplast.

A second group of spots in the EGTA fraction contains isoforms of aspartyl protease, which are of lower abundance in Cd-exposed plants. The proteins identified in these spots are predicted to be secreted (Table S2). Although no unambiguous function is established for secreted aspartyl proteases, they have been identified before, for instance in the pollen cell wall [42]. Functional studies have implicated them in the defence against biotic stresses [43] and the *Arabidopsis* homolog AED1 was recently proposed to be part of a homeostatic feedback mechanism regulating the systemic acquired resistance (SAR) response [44]. This link between SAR, a salicylic acid-regulated process, and the here-observed limited effect of long-term Cd exposure confirms previous studies, wherein salicylic acid application is shown to alleviate Cd-induced growth inhibition [45].

The only photosynthetic protein found with an increasing abundance is the photosystem I reaction centre subunit II (CaCl_2_ fraction spot 3075, Table S2). The position of this spot is, however, at too low a pI and molecular weight, providing a further indication for increasing protein degradation in the chloroplast.

### 3.2. Cd Influences the Abundance of Proteins Related to the Cell Wall Structure

Pectinesterase/pectinesterase inhibitor proteins are identified in three spots (Table S2, CaCl_2_ fraction spot 2175 and EGTA fraction spot 989, 1007). The abundance of these proteins decreases in Cd-exposed plants. PMEs and their inhibitor are expressed as a single polypeptide and get subsequently processed by cleavage between the inhibitor and active domain. Our MS data do not confirm that the inhibitor domain is cut from the PME domain. However, the position on the 2D gel matches with what is predicted for the active protein and are at the same position as those found in *M. sativa* stems [30]. In the latter study, cleavage between the inhibitor and the active domain was confirmed based on MS data, indicating that in leaves the inhibitor domain was also cleaved and thus the protein activated. However, the abundance of PME increased in *M. sativa* stems in response to long-term Cd exposure [30].

PME catalyses the demethylesterification of HG in plant cell walls. Those demethylated, acidic HG molecules form bridges between each other mediated by Ca^2+^ ions (egg-box structure) and confer rigidity to the cell wall [10,46]. Cadmium ions have the same charge (Cd^2+^) and can displace Ca^2+^ as cross linker in the pectin egg-box structure. During Cd exposure a higher abundance of PME was found in several studies and an enhanced activity demonstrated [14,16]. Changes in HG pattern in response to Cd have been investigated together with a preferential allocation of Cd in the cell wall [12,47,48]. Thus, the cell wall structure changes during Cd exposure and the sequestration of Cd in the cell wall protects the cytosol. In a recent study on the cell wall composition in *M. sativa* stems upon long-term Cd exposure, the most significant changes appeared in the pectin fraction towards a higher abundance of HG upon Cd exposure and an increased activity of PME in response to Cd was determined (Gutsch et al., submitted). This suggests a high demethylation degree, which creates binding sites for Cd and immobilize it in the cell wall. Opposite to what is observed in *M. sativa* stems [30], Cd exposure led to a decreasing abundance of PME in leaves, which would lead to a low demethylation degree of HG. The methylation degree of pectin has an influence on the cell wall structure and, furthermore, limits the accessibility for pectin-degrading enzymes, such as polygalacturonases [49]. In response to long-term Cd exposure, the structural changes in the pectin network of the leaf cell wall seem to be different from those anticipated in the stems of *M. sativa* in response to Cd [30] and an organ-specific influence of Cd on the cell wall can be assumed. Together with so-far-unpublished data (Gutsch et al., submitted), the structural changes, which are observed in the stems of *M. sativa*, promote the creation of binding sites for Cd in the stem cell wall as a direct response to the applied stress. In this matter, the leaf cell wall plays probably a minor role in the retention of Cd but more data on the structural changes would be needed to draw a more comprehensive conclusion.

Polygalacturonase-inhibiting protein 1 and polygalacturonase non-catalytic protein or β-subunit are involved in pectin degradation. Conflicting data on the function of the latter has been obtained. Overexpression of the polygalacturonase non-catalytic β-subunit in rice resulted in a decreased pectin content and a higher susceptibility to abiotic stress due to lower cell adhesion [50]. On the other hand, during fruit ripening in tomato, it limits the extent of pectin solubilisation and depolymerization [51]. In the present study, a lower abundance occurred in the *M. sativa* leaf subproteome enriched in cell wall proteins (Table 1), as was previously reported in *M. sativa* stems [30]. However, a quantitative change in the abundance of β-subunit of polygalacturonase is not necessarily correlated with a change in polygalacturonase activity [52]. The functional linkage between these two polygalacturonase subunits remains to be solved as contradictory data exist. Furthermore, polygalacturonase inhibitor showed a decreasing abundance in our study. It can be speculated that this decreased abundance is a cross reaction to the lower abundance of polygalacturonase non-catalytic protein in order to keep the polygalacturonase activity in the cell wall stable. So far, polygalacturonase inhibitor has only been described during the defence against pathogen attacks by inhibiting the fungal polygalacturonase [53,54]. Those proteins have a specific binding site to interact with pectin in the plant cell wall, which is furthermore influenced by methylesterification patterns in the pectin network [55]. Their function in the alteration of the plant cell wall structure in response to abiotic stress remains unclear.

Various peroxidase isoforms were highly abundant in the leaves of Cd-exposed *M. sativa* (Table 1). Previous studies on *M. sativa* stems [30] and poplar leaves gave similar results [56]. Peroxidases are involved in oxidation-reduction and lignification processes in the cell wall [57]. Using H_2_O_2_ molecules as a co-substrate, peroxidases catalyse the oxidation of monolignols, which then cross-link to form lignin [58] and increasing peroxidase activity was positively correlated with the degree of cell wall lignification [59]. Cadmium enhances H_2_O_2_ accumulation in plants and increased peroxidase abundance and activity, leading to cell wall lignification, cell wall stiffening and growth impairment [15,60,61]. Oxidative stress might also be responsible for the accumulation of carboxyl-terminal peptidase (Table 1). The protein contains two DUF domains (239 and 4409). The *Arabidopsis* homolog was found to be responsive to ROS and confers enhanced tolerance to oxidative stress [62]. The same protein was found to have a role in cell wall modification, influencing nutrient transport by modification of root endodermal barriers [63].

### 3.3. Enhanced Accumulation of Defence Proteins as a Response to Cd

Different defence related proteins were identified in *M. sativa* leaves (Table 1). Their increased accumulation due to Cd exposure underline the strong defence response of the plant, which had been reported before when plants were exposed to heavy metals [30,37,56,64]. Most prevalent, we identified different chitinase isoforms throughout all three fractions (Table S2), which is consistent with previous findings in *M. sativa* stems [30], where several chitinase isoforms increased in abundance upon long-term Cd-exposure. A study on different plant species exposed to different metals indicates that a metal-specific chitinase-expression profile may exist [65]. Although such metal-specific functions of chitinases remain uncertain, an increased abundance of chitinases has been proposed as a marker for the induction of a SAR-response. Despite the small impact of long-term Cd exposure on biomass production [30], it appears to have a significant negative impact on the presence of proteins classified as having a nutrient reserve function (Figure 2), which was also reported in *M. sativa* stems [30]. This may indicate that the plants are capable of maintaining growth under the conditions used in this study, but are not capable of establishing reserves. It must, however, be mentioned that multiple functions are attributed to these nutrient reserve proteins and that the observed decreased abundance may have other consequences [66].

## 4. Materials and Methods

### 4.1. Plant Material and Treatment

*M. sativa* (cv Giulia) seeds were inoculated with *Sinorhizobium meliloti* and sown in May 2015 on Cd-polluted soil (10 mg/kg soil DW added as CdSO_4_) and uncontaminated soil. The used soil was prepared as one batch (2/3 potting soil mixed with 1/3 sand (*w*/*w*)) before splitting in two conditions. For each condition 12 times 12 pots were planted. The plants were kept in the greenhouse until flowering stage was reached (July) and a first cut was done as during agricultural cultivation of *M. sativa*. Subsequently, plants were kept outside to avoid insect infestation. After a re-growing period till the pre-flowering stage was reached, plants were put back into the greenhouse for one more week before sampling was done on the 10th of September. No temperature or day cycle control was done during the experiment and no fertilizer was applied. A pool of leaves was sampled in four replicates for each condition and directly frozen in liquid nitrogen. All samples were kept at −80 °C until further use.

### 4.2. Cell Wall Protein Enrichment

Cell wall protein-enriched fractions were obtained as described elsewhere [25] by using subsequently three different buffers with increasing ionic strength to extract also tightly bound proteins. Following the extraction, all three protein fractions (CaCl_2_, ethylene glycol-*bis*(β-aminoethyl ether)-*N*,*N*,*N*′,*N*′-tetraacetic acid [EGTA], LiCl) were concentrated with Amicon Ultra 15 10 K (Millipore, Burlington, MA, USA) by centrifugation (4700× *g*, 4 °C) to an approximate volume of 200 µL. Subsequently, the ReadyPrep 2D Cleanup kit (Bio-Rad, Hercules, CA, USA) was used to desalt the samples following the manufacturer’s instruction. Cleaned samples were solubilized in labelling buffer (7 M urea, 2 M thiourea, 2% *w*/*v* 3-[(3-Cholamidopropyl)dimethylammonio]-1-propanesulfonate (CHAPS), 30 mM Tris) and the protein concentrations determined using the Bradford method (Bradford reagent, Bio-Rad).

### 4.3. Quantitative Protein Analysis and Identification

A 2D DIGE was undertaken to compare protein abundances between conditions in the three different fractions. Therefore, 50 µg of protein from each sample were labelled with either Cy3 or Cy5 and a dye swap was applied to avoid a possible effect of preferential labelling. An internal standard composed of 25 μg protein from each replicate (four biological replicates from control and cadmium, respectively) was labelled with Cy2 for each fraction (CaCl_2_, EGTA, LiCl). Labelled samples were mixed, 9 µL Servalyte pH 3–10 (Serva Electrophoresis GmbH, Heidelberg, Germany) and 2.7 µL Destreak Reagent (GE Healthcare, Chicago, IL, USA) were added. The volumes were adjusted with lysis buffer (7 M urea, 2 M thiourea, 4% *w*/*v* CHAPS) to 450 µL. Samples were loaded onto Immobiline™ DryStrip 3–10 NL, 24 cm (GE Healthcare, Chicago, IL, USA) overnight, followed by isoelectric focusing (IEF): (1) constant 100 V for 4 h; (2) linear gradient up to 1000 V for 4 h; (3) constant 1000 V for 5 h; (4) linear gradient up to 10,000 V for 6 h; and (5) constant 10,000 V until a total of 80,000 volt hours were reached. IEF-strips were equilibrated in equilibration buffer (Serva Electrophoresis GmbH) according to the manufacturer’s instructions. 2D HPE™ Large Gels NF-12.5% (Serva Electrophoresis GmbH) were used for the second dimension and were run on a HPE tower system following the manufacturer’s instruction. After fixation (15% ethanol *v*/*v*, 1% *w*/*v* citric acid), three images from each of the gels were acquired using different wavelengths for the different labelling dyes (Cy2 488 nm, Cy3 532 nm, Cy5 642 nm) (Typhoon FLA 9500, GE Healthcare). SameSpots software v4.5 (TotalLab, Newcastle upon Tyne, UK) was used for the relative quantitative image analysis. Since the same internal standard is run on each gel of a fraction, alignment and normalisation with the internal standard allows comparison of the spots between repetitions. All statistical analyses were automatically done by the software. A spot was chosen for protein identification if a treatment effect was reported (fold-change ≥ 1.2, ANOVA *p*-value ≤ 0.05, Table S3 for spot volumes), if the spot was matched on all replicates and after manual validation. 

Selected spots (Figure 3 and Figure S1) were picked with an Ettan Spot Picker (GE Healthcare) and digested prior to MS/MS analyses as described before [25]. Mass spectra were acquired with 5800 MALDI TOF/TOF (AB Sciex, Framingham, MA, USA). The ten most abundant peaks were automatically selected for fragmentation and spectra submitted to an in-house MASCOT server (Matrix Science, Available online: www.matrixscience.com) for database-dependent identifications. A first search was performed against the NCBInr database limited to *Viridiplantae* (3,334,509 sequences) and a second one against *M. sativa* sequences downloaded from the Samuel Roberts Noble website (Available online: plantgrn.noble.org/AGED (675,756 sequences, 304,231,576 residues)) [67]. The search parameters were as follows: mass tolerance 100 ppm, fragment mass tolerance 0.5 Da, cysteine carbamidomethylation as fixed modification, methionine oxidation, double oxidation of tryptophan, and tryptophan to kynurenine as variable modification. When at least two peptides passed the MASCOT-calculated 0.05 threshold score of 40, proteins were considered as identified. Additionally, if a high-quality spectrum was not matched to a protein, the interpretation was done manually and search parameters adjusted (semitryptic, single amino acid change, and post-translational modification) to increase the sequence coverage of identified proteins. After manual validation of the identifications, the subcellular location was predicted with the TargetP server using the standard search parameters (Available online: http://www.cbs.dtu.dk/services/TargetP) [68]. Only proteins with a predicted signal peptide have been considered as cell wall proteins as done in current literature [17,69]. To validate the predicted subcellular location, a second location prediction was undertaken using DeepLoc [70]. In some cases predictions were corrected after literature research. The Blast2Go software was used to gather information about the biological function of the identified proteins as well as the available literature.

## 5. Conclusions

*M. sativa* plants were exposed to Cd (10 mg/kg soil DW) in a long-term experiment and the leaf cell wall protein-enriched subproteome was analysed. In total, 212 identified proteins changed significantly in response to Cd and a major part of these identified proteins is involved in defence responses, underlining the importance of the general defence machinery in response to Cd and linking the observations in this study with knowledge on the SAR response. Cell wall proteins related to oxidation-reduction processes are highly abundant in Cd-exposed plants and might counteract the Cd-induced oxidative burst in the plant. Germin-like proteins, although classified as nutrient reserve, may also contribute. Interestingly, Cd provokes tissue-specific alterations in the pectin network of the cell wall in *M. sativa* leaves and stems [30]. The leaf cell wall seems to be less involved in the assumed cell wall-promoted binding of Cd as a protective mechanism.

About 18% of the identified proteins are targeted to the chloroplast and their relative abundance decreases upon Cd exposure concomitantly with an increase in chloroplastic, proteolytic proteins. Therefore, the increased protein degradation in the chloroplast confirms interference of Cd with the photosynthetic activity of plants. Nonetheless, Cd-exposed plants showed no difference in biomass production or in the growth at the moment of sampling in comparison to control plants, which suggests that the plants established a new metabolic steady-state during the long-term stress exposure. The important decrease in proteins with nutrient reserve function, however, indicates that the plants are weakened and may perform worse than control plants when exposed to secondary stresses.

## Figures and Tables

**Table 1:** Summary of all identified proteins in cell wall protein-enriched fractions from *M. sativa* leaves, which changed significantly in abundance after long-term Cd exposure. The table is based on all identifications provided in Table S2. The targeted location was predicted with TargetP. C: chloroplast; S: secretory pathway; /: any other location.

Protein Identification	NCBI Identification *	Nr. of Spots Wherein the Protein Was Identified	TargetP
**Lower Abundant in Cd-Exposed Plants**			
**Carbohydrate Metabolic Process**			
Sedoheptulose-1,7-bisphosphatase	gi|357461143	1	C
**Cell wall modification**			
Pectinesterase/pectinesterase inhibitor	gi|357504799	3	S
Polygalacturonase non-catalytic protein	gi|922335979	10	S
Polygalacturonase-inhibiting protein 1	gi|374634428	1	/
**Defence**			
Cystatin	gi|74058377	1	/
Nod factor-binding lectin-nucleotide phosphohydrolase	gi|357508587	4	S
Pathogenesis-related thaumatin family protein	gi|922367846	1	S
CAP, cysteine-rich secretory protein, antigen 5	gi|357446161	1	S
**Nutrient reserve**			
Auxin-binding protein ABP19a	gi|357513969	11	S
Germin-like protein subfamily 3 member 1	gi|502156424	1	S
Stem 28 kDa glycoprotein	gi|357513539	12	S
**Oxidation-reduction process**			
1-cys peroxiredoxin PER1	gi|922395795	1	C
**Photosynthesis**			
Chlorophyll a-b binding protein 2	gi|3293555	1	C
Oxygen-evolving enhancer protein	gi|922331371	6	C
Ribose-5-phosphate isomerase A	gi|357512271	4	C
Ribulose bisphosphate carboxylase small chain	gi|3914601	5	C
Photosystem I reaction centre subunit IV A	gi|922402507	1	C
Photosystem II oxygen-evolving enhancer protein	gi|922336891	1	C
**Proteolysis**			
Eukaryotic aspartyl protease family protein	gi|922379288	6	S
**Secondary metabolites**			
Lactoylglutathione lyase-like protein	gi|922388614	1	/
**Unknown**			
Plant/F18G18-200 protein	gi|922395263	6	S
**Higher Abundant in Cd-Exposed Plants**			
**Carbohydrate metabolic process**			
Glucan endo-1,3-β-glucosidase	gi|357474061	11	S
Glycoside hydrolase, family 17	gi|87240471	1	/
Glycoside hydrolase family 18 protein	gi|357454031	3	S
**Defence**			
Allergen Pru protein, putative	gi|922401927	7	S
Chitinase (Class Ib)/Hevein	gi|922329699	6	S
Chitinase/Hevein/PR-4/Wheatwin2	gi|922347233	10	S
Chitinase	gi|357443753	4	S
Class I chitinase	gi|1800141	5	S
Disease resistance response protein	gi|922325015	2	S or/
Pathogenesis-related protein 1	gi|548592	1	S
Pathogenesis-related thaumatin family protein	gi|922338021	4	S
Plant basic secretory protein (BSP) family protein	gi|922407517	2	S
Pre-hevein-like protein	gi|7381205	1	/
Stromal 70 kDa heat shock-related protein	gi|821595433	1	C
CAP, cysteine-rich secretory protein, antigen 5	gi|357446161	7	S
**Nutrient reserve**			
Auxin-binding protein ABP19a	gi|357513969	1	S
Rhicadhesin receptor	gi|357511665	2	S
**Oxidation-reduction process**			
Anionic peroxidase swpb3 protein	gi|922380311	1	S
Class III peroxidase	gi|357476371	10	S
Peroxidase	gi|537317	7	S
Peroxidase family protein	gi|357448431	1	S
Peroxidase1b	gi|971560	3	S
Peroxidase2	gi|13992528	10	S
Plastocyanin-like domain protein	gi|922335020	2	S
**Photosynthesis**			
Photosystem I reaction centre subunit II	gi|357480841	1	C
**Proteolysis**			
Carboxyl-terminal peptidase	gi|922336331	2	S
Eukaryotic aspartyl protease family protein	gi|922327497	14	C
Papain family cysteine protease	gi|357437715	2	S
Polyubiquitin	gi|695063425	6	/
Subtilisin-like serine protease	gi|922333118	1	S

* The given NCBI identification is representative, more than one identifier was assigned to the same protein. A complete protein identification list including all NCBI identification numbers is provided in Table S2.

## Supplementary Materials

Supplementary Materials can be found at http://www.mdpi.com/1422-0067/19/9/2498/s1. Table S1. Complete MASCOT protein identification data of picked spots (xls 819 KB). Table S2. The table contains all identified proteins from each fraction and their abundance change due to Cd exposure. Fold change and *p*-values were obtained using the SameSpots software (TotalLab). Functional classification of each protein was determined using Blast2Go. Subcellular localization was predicted with TargetP and DeepLoc (docx 37 KB). Figure S1. 2D DIGE images from the cell wall protein-enriched EGTA and LiCl fractions of *M. sativa* leaves (docx. 2.118 KB). Table S3. All spots and corresponding volumes (relative and normalized) are given as detected with SameSpots software (TotalLab) including statistical parameters automatically calculated by the software. Spots that were selected for identification are indicated (xlsx 536 KB).

## Abbreviations

2D DIGE	Two-dimensional difference gel electrophoresis
Ca	Calcium
Cd	Cadmium
DW	Dry weight
HG	Homogalacturonan
kDa	Kilo Dalton
MALDI	Matrix-assisted Laser Desorption/Ionization
MS	Mass spectrum
MW	Molecular weight
PCA	Principal component analysis
PME	Pectin methylesterase
ROS	Reactive oxygen species
RuBisCO	Ribulose bisphosphate carboxylase/oxidase
SAR	Systemic acquired resistance
TOF	Time of flight
