# Supplementary material for: Changes in the Proteome of Medicago sativa Leaves in Response to Long-Term Cadmium Exposure Using a Cell-Wall Targeted Approach
Source: Int J Mol Sci. 2018 Aug 24;19(9):2498. doi: 10.3390/ijms19092498 (PMC6165176; doi:10.3390/ijms19092498)
Supplement: Supplementary file 1 [file ijms-19-02498-s001.zip › FogireS1.pdf]

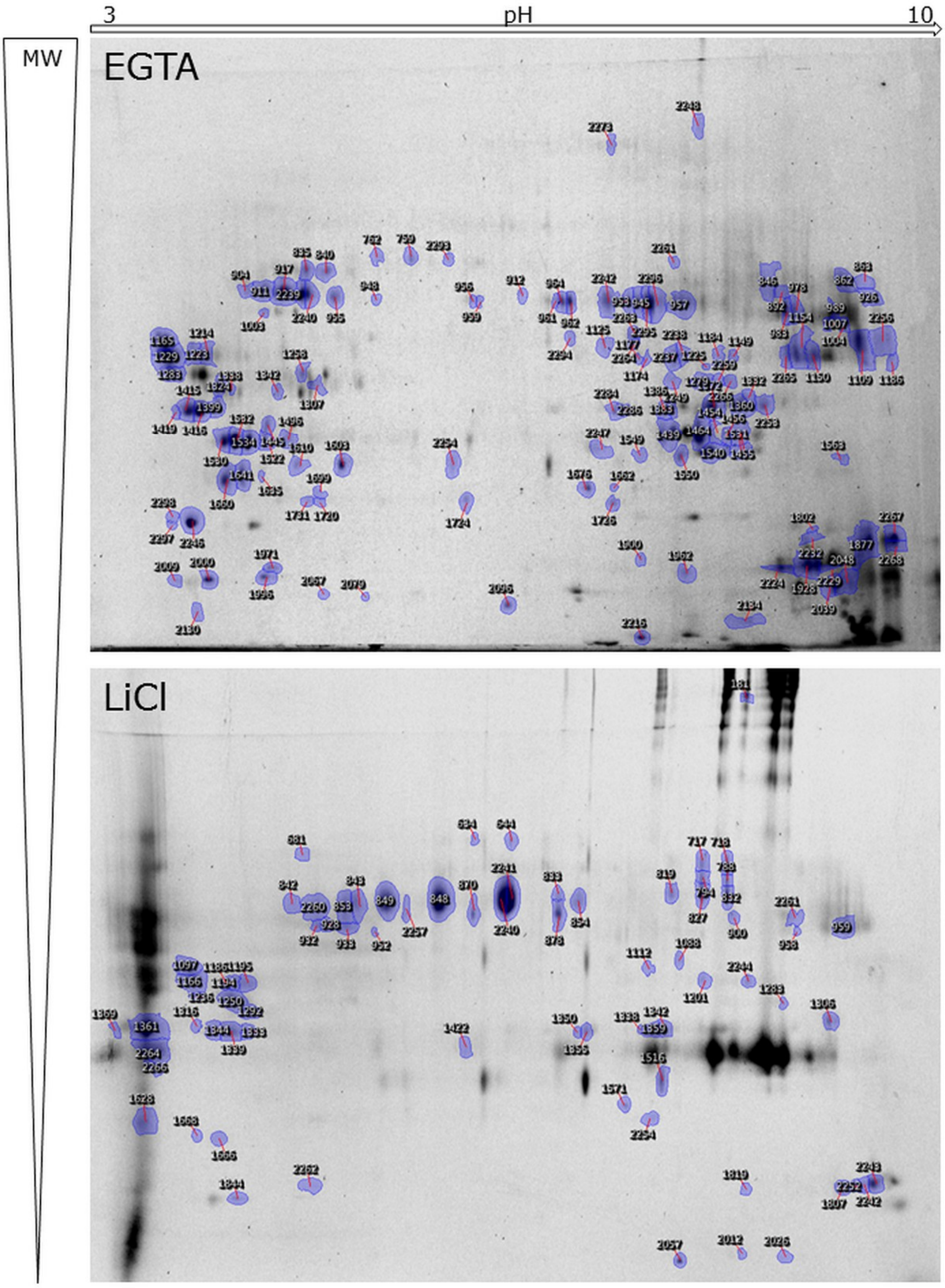

Figure S1. 2D DIGE from the cell wall protein-enriched EGTA and LiCl fractions of *M. sativa* leaves. Extractions were performed in four replicates. Proteins were pre-labelled with different CyDye to enable relative quantitative protein analysis. Labelled samples were loaded onto Immobiline™ DryStrip 3-10 NL, 24 cm (GE Healthcare) followed by migration on HPE™ Large Gel NF-12.5 % (Serva Electrophoresis GmbH). Indicated spots were selected for picking based on statistical parameters calculated by the SameSpots software (TotalLab). MW = molecular weight.
