# Supplementary material for: Changes in the Proteome of Medicago sativa Leaves in Response to Long-Term Cadmium Exposure Using a Cell-Wall Targeted Approach
Source: Int J Mol Sci. 2018 Aug 24;19(9):2498. doi: 10.3390/ijms19092498 (PMC6165176; doi:10.3390/ijms19092498)
Supplement: Supplementary file 1 [file ijms-19-02498-s001.zip › TableS2.pdf]

Table S2. Identified proteins in all three cell wall protein-enriched fractions ($CaCl_2$, EGTA, LiCl). The given spot numbers correspond to those detected on gel, which changed significantly in response to Cd exposure. The table only contains those spots in which a single protein was identified and were, therefore, considered for biological interpretation. The given gi- numbers correspond to NCBI protein database. Statistics were automatically done by the SameSpots software. The predicted subcellular localization was determined by TargetP online tool (http://www.cbs.dtu.dk/services/TargetP/). S: secretory pathway; C: chloroplast; M: mitochondrion; /: any other location. Information about the biological function were obtained with the Blast2Go software and literature. FC: Fold change; [a]: as a result of the prediction by DeepLoc, [b]:Type of protein, soluble or membrane, respectively indicated with Sol and Memb

# spot	Identification	Assigned protein	p-value	FC	Functional class	TargetP	Localisation [a]	Type [a.b]
**<u>$CaCl_2$ fraction</u>**								
Proteins of lower abundance in Cd-exposed plants								
2175	gi|357504821	pectinesterase/pectinesterase inhibitor (*Medicago truncatula*)	2.50E–02	1.8	cell wall modification	S	Lysosome/Vacuole	Sol
1771	gi|922335979	polygalacturonase non-catalytic protein (*Medicago truncatula*)	1.80E–02	1.9	cell wall modification	S	Extracellular	Sol
3105	gi|357446161	CAP, cysteinE–rich secretory protein, antigen 5 (*Medicago truncatula*)	1.70E–02	1.9	defence	S	Lysosome/Vacuole	Sol
239	gi|357513969	auxin-binding protein ABP19a (*Medicago truncatula*)	1.30E–02	1.3	nutrient reserve	S	Extracellular	Sol
2689	gi|357513969	auxin-binding protein ABP19a (*Medicago truncatula*)	6.00E–03	1.3	nutrient reserve	S	Extracellular	Sol
2690	gi|357513969	auxin-binding protein ABP19a (*Medicago truncatula*)	1.00E–03	1.5	nutrient reserve	S	Extracellular	Sol
2692	gi|357513969	auxin-binding protein ABP19a (*Medicago truncatula*)	2.77E–04	1.4	nutrient reserve	S	Extracellular	Sol
2783	gi|922390231	auxin-binding protein ABP19a (*Medicago truncatula*)	3.30E–02	1.6	nutrient reserve	S	Extracellular	Sol
2790	gi|922390231	auxin-binding protein ABP19a (*Medicago truncatula*)	3.00E–03	1.3	nutrient reserve	S	Extracellular	Sol
2786	gi|11133884	Oxygen-evolving enhancer protein 2	5.00E–03	1.7	photosynthesis	C	Plastid	Memb
2863	gi|922378759	oxygen-evolving enhancer protein 2-1 (*Medicago truncatula*)	1.20E–02	1.8	photosynthesis	C	Plastid	Memb

3645	gi|922378761	oxygen-evolving enhancer protein 2-1 (*Medicago truncatula*)	4.70E–02	1.7	photosynthesis	C	Plastid	Memb
3213	gi|3914601	Ribulose bisphosphate carboxylase small chain,	1.00E–02	2.2	photosynthesis	C	Plastid	Memb
3214	gi|3914601	Ribulose bisphosphate carboxylase small chain,	5.79E–04	1.7	photosynthesis	C	Plastid	Memb
3219	gi|3914601	Ribulose bisphosphate carboxylase small chain,	2.00E–02	1.5	photosynthesis	C	Plastid	Memb
3231	gi|3914601	Ribulose bisphosphate carboxylase small chain,	2.10E–02	2.2	photosynthesis	C	Plastid	Memb
3211	gi|3914601	ribulosE–1,5-bisphosphate carboxylase small subunit (*Medicago sativa*)	2.40E–02	1.7	photosynthesis	C	Plastid	Memb
3561	gi|3914601	ribulosE–1,5-bisphosphate carboxylase small subunit (*Medicago sativa*)	1.00E–03	1.7	photosynthesis	C	Plastid	Memb
2395	gi|922336891	photosystem II oxygen-evolving enhancer protein (*Medicago truncatula*)	4.00E–02	3.6	photosynthesis	C	Plastid	Memb
3558	gi|922395263	plant/F18G18-200 protein (*Medicago truncatula*)	4.00E–02	1.3	unknown function	S	Extracellular	Sol
3611	gi|922395263	plant/F18G18-200 protein (*Medicago truncatula*)	6.00E–03	1.8	unknown function	S	Extracellular	Sol
3672	gi|922395263	plant/F18G18-200 protein (*Medicago truncatula*)	2.00E–03	1.6	unknown function	S	Extracellular	Sol
Proteins of higher abundance in Cd-exposed plants								
3620	gi|87240471	Glycoside hydrolase, family 17 (*Medicago truncatula*)	4.00E–03	1.8	carbohydrate metabolic process	/	Lysosome/Vacuole	Sol
2280	gi|357474069	glucan endo-1,3-beta-glucosidase (*Medicago truncatula*)	6.66E–04	3.2	carbohydrate metabolic process	S	Extracellular	Sol
2420	gi|357454031	glycoside hydrolase family 18 protein (*Medicago truncatula*)	1.80E–02	1.9	carbohydrate metabolic process	S	Lysosome/Vacuole	Sol
3547	gi|922383650	glycoside hydrolase family 18 protein (*Medicago truncatula*)	7.04E–04	2.4	carbohydrate metabolic process	S	Lysosome/Vacuole	Sol

3124	gi|357449105	diseasE–resistance response protein (*Medicago truncatula*)	1.58E–04	3.9	defence	/	Cytoplasm	Sol
2402	gi|821595433	stromal 70 kDa heat shock-related protein, chloroplastic (*Cucumis sativus*)	3.00E–02	1.6	defence	C	Plastid	Sol
2236	gi|922401927	allergen Pru protein, putative (*Medicago truncatula*)	1.00E–03	3.3	defence	S	Extracellular	Sol
2915	gi|922347233	Chitinase / Hevein / PR-4 / Wheatwin2 (*Medicago truncatula*)	2.00E–03	1.8	Defence	S	Extracellular	Sol
3218	gi|922403681	Chitinase / Hevein / PR-4 / Wheatwin2 (*Medicago truncatula*)	5.00E–03	3	Defence	S	Extracellular	Sol
2508	gi|357443753	chitinase (*Medicago truncatula*)	5.00E–03	3.9	Defence	S	Extracellular	Sol
2525	gi|228204925	chitinase class III-1 (*Medicago sativa*)	3.20E–02	1.4	Defence	S	Extracellular	Sol
3489	gi|548592	Pathogenesis-related protein 1;	6.51E–05	2.4	defence	S	Lysosome/Vacuole	Sol
3028	gi|357446161	CAP, cysteinE–rich secretory protein, antigen 5 (*Medicago truncatula*)	3.61E–04	3.1	defence	S	Lysosome/Vacuole	Sol
3030	XP003593358	CAP, cysteinE–rich secretory protein, antigen 5 (*Medicago truncatula*)	5.75E–05	3.1	defence	S	Lysosome/Vacuole	Sol
3031	gi|357446161	CAP, cysteinE–rich secretory protein, antigen 5 (*Medicago truncatula*)	2.96E–04	2.6	defence	S	Lysosome/Vacuole	Sol
3034	gi|357446351	CAP, cysteinE–rich secretory protein, antigen 5 (*Medicago truncatula*)	1.50E–02	1.4	defence	S	Lysosome/Vacuole	Sol
3037	gi|357446161	CAP, cysteinE–rich secretory protein, antigen 5 (*Medicago truncatula*)	2.20E–02	1.7	defence	S	Lysosome/Vacuole	Sol
3081	gi|357446161	CAP, cysteinE–rich secretory protein, antigen 5 (*Medicago truncatula*)	4.70E–04	2	defence	S	Lysosome/Vacuole	Sol
3095	gi|357446161	CAP, cysteinE–rich secretory protein, antigen 5 (*Medicago truncatula*)	4.60E–02	1.4	defence	S	Lysosome/Vacuole	Sol
2339	gi|357476371	class III peroxidase (*Medicago truncatula*)	2.39E–04	2	oxidation-reduction process	S	Extracellular	Sol

2349	gi|357476371	class III peroxidase (*Medicago truncatula*)	3.00E–03	1.9	oxidation-reduction process	S	Extracellular	Sol
2357	gi|357476371	class III peroxidase (*Medicago truncatula*)	3.21E–04	2	oxidation-reduction process	S	Extracellular	Sol
2359	gi|357476371	class III peroxidase (*Medicago truncatula*)	6.35E–04	1.9	oxidation-reduction process	S	Extracellular	Sol
2371	gi|357476371	class III peroxidase (*Medicago truncatula*)	1.20E–02	1.5	oxidation-reduction process	S	Extracellular	Sol
3519	gi|357476371	class III peroxidase (*Medicago truncatula*)	7.62E–06	2	oxidation-reduction process	S	Extracellular	Sol
3663	gi|357476371	class III peroxidase (*Medicago truncatula*)	1.20E–04	1.9	oxidation-reduction process	S	Extracellular	Sol
2342	gi|971564	peroxidase2 (*Medicago sativa*)	2.00E–03	1.8	oxidation-reduction process	S	Extracellular	Sol
2356	gi|13992528	peroxidase2 (*Medicago sativa*)	1.27E–04	2.1	oxidation-reduction process	S	Extracellular	Sol
2372	gi|13992528	peroxidase2 (*Medicago sativa*)	7.41E–04	1.8	oxidation-reduction process	S	Extracellular	Sol
3535	gi|971564	peroxidase2 (*Medicago sativa*)	2.61E–04	1.9	oxidation-reduction process	S	Extracellular	Sol
3536	gi|13992528	peroxidase2 (*Medicago sativa*)	2.49E–04	1.8	oxidation-reduction process	S	Extracellular	Sol
3617	gi|13992528	peroxidase2 (*Medicago sativa*)	7.00E–03	2	oxidation-reduction process	S	Extracellular	Sol
3636	gi|13992528	peroxidase2 (*Medicago sativa*)	1.11E–05	2	oxidation-reduction process	S	Extracellular	Sol
3637	gi|13992528	peroxidase2 (*Medicago sativa*)	2.30E–02	1.7	oxidation-reduction process	S	Extracellular	Sol
3664	gi|13992528	peroxidase2 (*Medicago sativa*)	9.39E–06	2.1	oxidation-reduction process	S	Extracellular	Sol
3158	gi|922335020	plastocyanin-like domain protein (*Medicago truncatula*)	3.80E–02	1.4	oxidation-reduction process	S	Cell Memb	Memb
3075	gi|357480841	photosystem I reaction center subunit II (*Medicago truncatula*)	2.00E–03	2.4	photosythesis	C	Plastid	Sol

1922	gi|922327497	eukaryotic aspartyl protease family protein (*Medicago truncatula*)	1.00E–03	2.8	proteolysis	C	Extracellular	Sol
1938	gi|922327497	eukaryotic aspartyl protease family protein (*Medicago truncatula*)	3.00E–03	3.3	proteolysis	C	Extracellular	Sol
3541	gi|922327497	eukaryotic aspartyl protease family protein (*Medicago truncatula*)	1.40E–02	2.1	proteolysis	C	Extracellular	Sol
3595	gi|922327497	eukaryotic aspartyl protease family protein (*Medicago truncatula*)	1.00E–02	1.9	proteolysis	C	Extracellular	Sol
2699	gi|922336331	carboxyl-terminal peptidase (*Medicago truncatula*)	9.00E–03	1.6	proteolysis	S	Extracellular	Sol
2374	gi|357437715	papain family cysteine protease (*Medicago truncatula*)	1.90E–02	1.4	proteolysis	S	Lysosome/Vacuole	Sol

# spot	Identification	Assigned protein	p-value	FC	Functional class	TargetP	Localisation [a]	Type [a.b]
**EGTA fraction**								
Proteins of lower abundance in Cd-exposed plants								
1003	gi|357461143	sedoheptulosE–1,7-bisphosphatase (*Medicago truncatula*)	1.90E–02	2.1	carbohydrate metabolic process	C	Plastid	Sol
892	gi|374634428	polygalacturonasE–inhibiting protein 1, partial (*Medicago sativa* subsp. x varia)	3.89E–04	1.6	cell wall modification	/	Extracellular	Sol
989	gi|357504799	pectinesterase/pectinesterase inhibitor (*Medicago truncatula*)	2.00E–03	3.2	cell wall modification	S	Lysosome/Vacuole	Sol
1007	gi|357504799	pectinesterase/pectinesterase inhibitor (*Medicago truncatula*)	2.00E–03	3.5	cell wall modification	S	Lysosome/Vacuole	Sol
2096	gi|74058377	cystatin (*Medicago sativa*)	1.00E–03	1.9	defence	/	Cytoplasm	Sol
1439	gi|922367846	pathogenesis-related thaumatin family protein (*Medicago truncatula*)	3.00E–03	1.9	defence	S	Extracellular	Sol
759	gi|357508587	Nod factor-binding lectin-nucleotide phosphohydrolase (*Medicago truncatula*)	4.70E–02	1.8	Defence	S	Extracellular	Sol
762	gi|922345135	Nod factor-binding lectin-nucleotide phosphohydrolase (*Medicago truncatula*)	2.40E–02	1.7	Defence	S	Extracellular	Sol

835	gi|922339971	Nod factor-binding lectin-nucleotide phosphohydrolase (*Medicago truncatula*)	4.00E–02	1.5	Defence	S	Extracellular	Sol
2293	gi|922345135	Nod factor-binding lectin-nucleotide phosphohydrolase (*Medicago truncatula*)	5.00E–03	1.9	Defence	S	Extracellular	Sol
1454	gi|357513969	auxin-binding protein ABP19a (*Medicago truncatula*)	2.00E–03	2	nutrient reserve	S	Extracellular	Sol
1455	gi|357513969	auxin-binding protein ABP19a (*Medicago truncatula*)	1.00E–03	2.5	nutrient reserve	S	Extracellular	Sol
1456	gi|357513969	auxin-binding protein ABP19a (*Medicago truncatula*)	4.00E–03	2.3	nutrient reserve	S	Extracellular	Sol
2254	gi|922390231	auxin-binding protein ABP19a (*Medicago truncatula*)	3.00E–03	1.4	nutrient reserve	S	Extracellular	Sol
1464	gi|502156424	germin-like protein subfamily 3 member 1 (*Cicer arietinum*)	8.36E–05	2.8	nutrient reserve	S	Extracellular	Sol
1383	gi|357513539	stem 28 kDa glycoprotein (*Medicago truncatula*)	1.60E–02	2.1	nutrient reserve	S	Extracellular	Sol
1386	gi|357513539	stem 28 kDa glycoprotein (*Medicago truncatula*)	2.30E–02	1.8	nutrient reserve	S	Extracellular	Sol
1726	gi|357513539	stem 28 kDa glycoprotein (*Medicago truncatula*)	5.00E–03	2	nutrient reserve	S	Extracellular	Sol
1641	gi|922395795	1-cys peroxiredoxin PER1 (*Medicago truncatula*)	2.00E–03	2.3	oxidation-reduction process	C	Plastid	Sol
1496	gi|3293555	Chlorophyll a-b binding protein 2, (*Populus euphratica*)	8.00E–03	1.7	photosynthesis	C	Plastid	Memb
2048	gi|922331371	oxygen-evolving enhancer protein (*Medicago truncatula*)	1.00E–03	2.4	photosynthesis	C	Plastid	Memb
1610	gi|502127023	oxygen-evolving enhancer protein 2, (*Cicer arietinum*)	3.50E–02	3.7	photosynthesis	C	Plastid	Memb
1603	gi|922378761	oxygen-evolving enhancer protein 2-1 (*Medicago truncatula*)	2.60E–02	5.9	photosynthesis	C	Plastid	Memb
1522	gi|357512271	ribosE–5-phosphate isomerase A (*Medicago truncatula*)	1.00E–03	2.5	photosynthesis	C	Plastid	Sol
1530	gi|357512271	ribosE–5-phosphate isomerase A (*Medicago truncatula*)	2.31E–05	3.1	photosynthesis	C	Plastid	Sol
1532	gi|357512271	ribosE–5-phosphate isomerase A (*Medicago truncatula*)	5.27E–04	4	photosynthesis	C	Plastid	Sol

1534	gi|357512271	ribosE–5-phosphate isomerase A (*Medicago truncatula*)	1.46E–04	5.3	photosynthesis	C	Plastid	Sol
2267	gi|922402507	photosystem I reaction center subunit IV A (*Medicago truncatula*)	2.10E–02	2.9	photosythesis	C	Plastid	Memb
956	gi|922379288	eukaryotic aspartyl protease family protein (*Medicago truncatula*)	1.30E–02	1.5	proteolysis	S	Extracellular	Sol
961	gi|922379284	eukaryotic aspartyl protease family protein (*Medicago truncatula*)	2.00E–03	1.9	proteolysis	S	Extracellular	Sol
962	gi|922379288	eukaryotic aspartyl protease family protein (*Medicago truncatula*)	5.00E–03	1.8	proteolysis	S	Extracellular	Sol
964	gi|922379288	eukaryotic aspartyl protease family protein (*Medicago truncatula*)	7.63E–04	2	proteolysis	S	Extracellular	Sol
978	gi|357448247	eukaryotic aspartyl protease family protein (*Medicago truncatula*)	4.00E–03	1.5	proteolysis	S	Extracellular	Sol
983	gi|357448247	eukaryotic aspartyl protease family protein (*Medicago truncatula*)	7.00E–03	1.7	proteolysis	S	Extracellular	Sol
1258	gi|922388614	lactoylglutathione lyasE–like protein (*Medicago truncatula*)	6.00E–03	2.3	secondary metabolite metabolism	/	Cytoplasm	Sol
2242	gi|922395263	plant/F18G18-200 protein (*Medicago truncatula*)	2.00E–03	2	unknown function	S	Extracellular	Sol
2295	gi|922395263	plant/F18G18-200 protein (*Medicago truncatula*)	1.00E–03	2.3	unknown function	S	Extracellular	Sol
2296	gi|922395263	plant/F18G18-200 protein (*Medicago truncatula*)	1.00E–03	2.5	unknown function	S	Extracellular	Sol
Proteins of higher abundance in Cd-exposed plants								
1996	gi|7381205	prE–hevein-like protein (*Pisum sativum*)	5.26E–05	2.3	defence	/	Extracellular	Sol
2216	gi|695063425	polyubiquitin (*Musa acuminata* subsp. *malaccensis*)	1.80E–02	1.5	proteolysis	/	Cytoplasm	Sol
2134	gi|33327284	polyubiquitin 1 (*Phaseolus vulgaris*)	3.30E–02	1.5	proteolysis	/	Nucleus	Sol
1900	gi|695062350	polyubiquitin-like, partial (*Musa acuminata* subsp. *malaccensis*)	1.10E–02	1.6	proteolysis	/	Nucleus	Sol
904	gi|922327497	eukaryotic aspartyl protease family protein (*Medicago truncatula*)	7.00E–03	2.2	proteolysis	C	Extracellular	Sol
911	gi|922327497	eukaryotic aspartyl protease family protein (*Medicago truncatula*)	1.50E–02	2.2	proteolysis	C	Extracellular	Sol

917	gi|922327497	eukaryotic aspartyl protease family protein (*Medicago truncatula*)	2.90E–02	2.1	proteolysis	C	Extracellular	Sol
948	gi|922327497	eukaryotic aspartyl protease family protein (*Medicago truncatula*)	1.80E–02	1.5	proteolysis	C	Extracellular	Sol
955	gi|922327497	eukaryotic aspartyl protease family protein (*Medicago truncatula*)	5.00E–03	2.5	proteolysis	C	Extracellular	Sol
2239	gi|922327497	eukaryotic aspartyl protease family protein (*Medicago truncatula*)	2.40E–02	2.2	proteolysis	C	Extracellular	Sol
2240	gi|922327497	eukaryotic aspartyl protease family protein (*Medicago truncatula*)	1.60E–02	2.2	proteolysis	C	Extracellular	Sol
1174	gi|357474061	glucan endo-1,3-beta-glucosidase (*Medicago truncatula*)	2.00E–03	2.5	carbohydrate metabolic process	S	Extracellular	Sol
1177	gi|922389309	glucan endo-1,3-beta-glucosidase (*Medicago truncatula*)	1.00E–03	2.1	carbohydrate metabolic process	/	Cytoplasm	Sol
1184	gi|922389309	glucan endo-1,3-beta-glucosidase (*Medicago truncatula*)	3.00E–03	2	carbohydrate metabolic process	/	Cytoplasm	Sol
1214	gi|357474069	glucan endo-1,3-beta-glucosidase (*Medicago truncatula*)	3.10E–02	1.9	carbohydrate metabolic process	S	Extracellular	Sol
1223	gi|357474069	glucan endo-1,3-beta-glucosidase (*Medicago truncatula*)	4.00E–03	2	carbohydrate metabolic process	S	Extracellular	Sol
2237	gi|922389309	glucan endo-1,3-beta-glucosidase (*Medicago truncatula*)	1.00E–03	2.4	carbohydrate metabolic process	/	Cytoplasm	Sol
2238	gi|357474069	glucan endo-1,3-beta-glucosidase (*Medicago truncatula*)	1.00E–03	2.4	carbohydrate metabolic process	S	Extracellular	Sol
1165	gi|922401927	allergen Pru protein, putative (*Medicago truncatula*)	1.00E–03	3.6	defence	S	Extracellular	Sol
1229	gi|922401937	allergen Pru protein, putative (*Medicago truncatula*)	5.91E–04	3.9	defence	S	Extracellular	Sol
1283	gi|922401937	allergen Pru protein, putative (*Medicago truncatula*)	2.00E–03	3	defence	S	Extracellular	Sol
1125	gi|922329699	Chitinase (Class Ib) / Hevein (*Medicago truncatula*)	8.12E–04	2.3	Defence	S	Lysosome/Vacuole	Sol
2263	gi|922329699	Chitinase (Class Ib) / Hevein (*Medicago truncatula*)	2.30E–02	1.8	Defence	S	Lysosome/Vacuole	Sol
2264	gi|922329699	Chitinase (Class Ib) / Hevein (*Medicago truncatula*)	8.00E–03	1.8	Defence	S	Lysosome/Vacuole	Sol

2294	gi|922329699	Chitinase (Class Ib) / Hevein (*Medicago truncatula*)	9.00E–03	1.7	Defence	S	Lysosome/Vacuole	Sol
1676	gi|922347233	Chitinase / Hevein / PR-4 / Wheatwin2 (*Medicago truncatula*)	3.50E–02	1.5	Defence	S	Extracellular	Sol
2000	gi|922403681	Chitinase / Hevein / PR-4 / Wheatwin2 (*Medicago truncatula*)	2.34E–04	3.3	Defence	S	Extracellular	Sol
2224	gi|922403683	Chitinase / Hevein / PR-4 / Wheatwin2 (*Medicago truncatula*)	1.00E–03	2.3	Defence	S	Extracellular	Sol
1399	gi|357443753	chitinase (*Medicago truncatula*)	4.25E–04	3	Defence	S	Extracellular	Sol
1416	gi|357443753	chitinase (*Medicago truncatula*)	2.00E–03	3.1	Defence	S	Extracellular	Sol
1419	gi|357443753	chitinase (*Medicago truncatula*)	3.20E–04	3.6	Defence	S	Extracellular	Sol
1360	gi|228204925	chitinase class III-1 (*Medicago sativa*)	7.63E–04	1.9	Defence	S	Extracellular	Sol
1372	gi|228204925	chitinase class III-1 (*Medicago sativa*)	2.00E–03	2.4	Defence	S	Extracellular	Sol
2253	gi|228204925	chitinase class III-1 (*Medicago sativa*)	6.27E–04	2	Defence	S	Extracellular	Sol
1225	gi|1800141	class I chitinase (*Medicago sativa*)	3.00E–03	1.9	Defence	S	Lysosome/Vacuole	Sol
1279	gi|1800141	class I chitinase (*Medicago sativa*)	1.00E–03	2.2	Defence	S	Lysosome/Vacuole	Sol
2249	gi|1800141	class I chitinase (*Medicago sativa*)	6.00E–03	1.8	Defence	S	Lysosome/Vacuole	Sol
2259	gi|1800141	class I chitinase (*Medicago sativa*)	3.00E–03	1.9	Defence	S	Lysosome/Vacuole	Sol
2266	gi|1800141	class I chitinase (*Medicago sativa*)	1.10E–02	1.8	Defence	S	Lysosome/Vacuole	Sol
912	gi|922325015	disease resistance response protein (*Medicago truncatula*)	4.09E–04	2	defence	S	Lysosome/Vacuole	Sol
1531	gi|922338021	pathogenesis-related thaumatin family protein (*Medicago truncatula*)	7.61E–04	1.7	defence	S	Extracellular	Sol
1540	gi|922338021	pathogenesis-related thaumatin family protein (*Medicago truncatula*)	2.00E–03	2.4	defence	S	Extracellular	Sol
2246	gi|922338023	pathogenesis-related thaumatin family protein (*Medicago truncatula*)	2.00E–03	4.9	defence	S	Extracellular	Sol
1549	gi|922407517	plant basic secretory protein (BSP) family protein (*Medicago truncatula*)	4.00E–03	1.8	defence	S	Extracellular	Sol
1550	gi|922407517	plant basic secretory protein (BSP) family protein (*Medicago truncatula*)	4.00E–03	2	defence	S	Extracellular	Sol
1332	gi|357511665	rhicadhesin receptor (*Medicago truncatula*)	2.00E–03	2.3	nutrient reserve	S	Extracellular	Sol

926	gi|922380311	anionic peroxidase swpb3 protein (*Medicago truncatula*)	2.90E–02	1.8	oxidation-reduction process	S	Extracellular	Sol
1109	gi|357491415	class III peroxidase (*Medicago truncatula*)	1.20E–02	1.6	oxidation-reduction process	S	Extracellular	Sol
1186	gi|357491415	class III peroxidase (*Medicago truncatula*)	2.50E–02	1.8	oxidation-reduction process	S	Extracellular	Sol
2256	gi|357491415	class III peroxidase (*Medicago truncatula*)	1.20E–02	1.8	oxidation-reduction process	S	Extracellular	Sol
1150	gi|13992528	peroxidase2 (*Medicago sativa*)	1.10E–02	2	oxidation-reduction process	S	Extracellular	Sol
2232	gi|922335020	plastocyanin-like domain protein (*Medicago truncatula*)	3.60E–02	1.4	oxidation-reduction process	S	Cell Memb	Memb
1445	gi|922336321	carboxyl-terminal peptidase (*Medicago truncatula*)	4.00E–03	1.7	proteolysis	S	Cell Memb	Memb
1971	gi|357437719	papain family cysteine protease (*Medicago truncatula*)	7.00E–03	1.9	proteolysis	S	Lysosome/Vacuole	Sol
2273	gi|922333118	subtilisin-like serine protease (*Medicago truncatula*)	3.20E–02	1.4	proteolysis	S	Extracellular	Sol

**# spot**	**Identification**	**Assigned protein**	**p-value**	**FC**	**Functional class**	**TargetP**	**Localisation [a]**	**Type [a.b]**
				**LiCl fraction**				
Proteins of lower abundance in Cd-exposed plants								
717	gi|922335979	polygalacturonase non-catalytic protein (*Medicago truncatula*)	2.60E–02	1.5	cell wall modification	S	Extracellular	Sol
718	gi|922335979	polygalacturonase non-catalytic protein (*Medicago truncatula*)	1.80E–02	1.6	cell wall modification	S	Extracellular	Sol
788	gi|922335981	polygalacturonase non-catalytic protein (*Medicago truncatula*)	2.10E–02	1.8	cell wall modification	S	Extracellular	Sol
794	gi|922335979	polygalacturonase non-catalytic protein (*Medicago truncatula*)	2.20E–02	1.6	cell wall modification	S	Extracellular	Sol
819	gi|922335979	polygalacturonase non-catalytic protein (*Medicago truncatula*)	8.00E–03	1.6	cell wall modification	S	Extracellular	Sol
827	gi|922335979	polygalacturonase non-catalytic protein (*Medicago truncatula*)	5.00E–03	1.9	cell wall modification	S	Extracellular	Sol

832	gi|922335979	polygalacturonase non-catalytic protein (*Medicago truncatula*)	6.00E–03	2.1	cell wall modification	S	Extracellular	Sol
870	gi|922335981	polygalacturonase non-catalytic protein (*Medicago truncatula*)	4.25E–05	4.3	cell wall modification	S	Extracellular	Sol
878	gi|922335981	polygalacturonase non-catalytic protein (*Medicago truncatula*)	2.50E–06	4.7	cell wall modification	S	Extracellular	Sol
181	gi|357513969	auxin-binding protein ABP19a (*Medicago truncatula*)	1.70E–02	1.3	nutrient reserve	S	Extracellular	Sol
1338	gi|357513539	stem 28 kDa glycoprotein (*Medicago truncatula*)	4.30E–02	1.5	nutrient reserve	S	Extracellular	Sol
1342	gi|357513539	stem 28 kDa glycoprotein (*Medicago truncatula*)	1.60E–02	1.7	nutrient reserve	S	Extracellular	Sol
1350	gi|357513539	stem 28 kDa glycoprotein (*Medicago truncatula*)	4.00E–03	1.7	nutrient reserve	S	Extracellular	Sol
1355	gi|357513539	stem 28 kDa glycoprotein (*Medicago truncatula*)	1.40E–02	1.7	nutrient reserve	S	Extracellular	Sol
1359	gi|357513539	stem 28 kDa glycoprotein (*Medicago truncatula*)	2.19E–04	1.5	nutrient reserve	S	Extracellular	Sol
1361	gi|357513539	stem 28 kDa glycoprotein (*Medicago truncatula*)	7.00E–03	2.1	nutrient reserve	S	Extracellular	Sol
1369	gi|357513539	stem 28 kDa glycoprotein (*Medicago truncatula*)	3.00E–03	2.3	nutrient reserve	S	Extracellular	Sol
1628	gi|357513539	stem 28 kDa glycoprotein (*Medicago truncatula*)	1.10E–02	1.4	nutrient reserve	S	Extracellular	Sol
2254	gi|357513539	stem 28 kDa glycoprotein (*Medicago truncatula*)	7.00E–03	2.1	nutrient reserve	S	Extracellular	Sol
Proteins of higher abundance in Cd-exposed plants								
2012	gi|922395829	pentameric polyubiquitin (*Medicago truncatula*)	3.90E–02	1.9	proteolysis	/	Cytoplasm	Sol
2026	gi|732558989	polyubiquitin 11, partial (*Pinus massoniana*)	3.00E–02	1.9	proteolysis	/	Nucleus	Sol
2057	gi|695062350	polyubiquitin-like, partial (*Musa acuminata* subsp. *malaccensis*)	7.00E–03	1.9	proteolysis	/	Nucleus	Sol
928	gi|922327497	eukaryotic aspartyl protease family protein (*Medicago truncatula*)	8.00E–03	2	proteolysis	C	Extracellular	Sol
933	gi|922327497	eukaryotic aspartyl protease family protein (*Medicago truncatula*)	2.00E–03	2	proteolysis	C	Extracellular	Sol

952	gi|922327497	eukaryotic aspartyl protease family protein (*Medicago truncatula*)	1.70E–02	2.4	proteolysis	C	Extracellular	Sol
1194	gi|357474069	glucan endo-1,3-beta-glucosidase (*Medicago truncatula*)	1.20E–02	2.6	carbohydrate metabolic process	S	Extracellular	Sol
1195	XP_00360731 9	glucan endo-1,3-beta-glucosidase (*Medicago truncatula*)	2.00E–03	2.5	carbohydrate metabolic process	S	Extracellular	Sol
1201	gi|357474069	glucan endo-1,3-beta-glucosidase (*Medicago truncatula*)	1.00E–03	3.4	carbohydrate metabolic process	S	Extracellular	Sol
1292	gi|357454031	glycoside hydrolase family 18 protein (*Medicago truncatula*)	3.00E–03	2.8	carbohydrate metabolic process	S	Lysosome/Vacuole	Sol
1097	gi|922401927	allergen Pru protein, putative (*Medicago truncatula*)	7.09E–06	4.5	defence	S	Extracellular	Sol
1166	gi|922401937	allergen Pru protein, putative (*Medicago truncatula*)	8.18E–06	4.8	defence	S	Extracellular	Sol
1236	gi|922401927	allergen Pru protein, putative (*Medicago truncatula*)	1.00E–03	4	defence	S	Extracellular	Sol
1088	gi|922329699	Chitinase (Class Ib) / Hevein (*Medicago truncatula*)	1.90E–02	2.2	Defence	S	Lysosome/Vacuole	Sol
1112	gi|922329699	Chitinase (Class Ib) / Hevein (*Medicago truncatula*)	7.00E–03	2.7	Defence	S	Lysosome/Vacuole	Sol
1571	gi|922347233	Chitinase / Hevein / PR-4 / Wheatwin2 (*Medicago truncatula*)	7.00E–03	2.4	Defence	S	Extracellular	Sol
1807	gi|922403683	Chitinase / Hevein / PR-4 / Wheatwin2 (*Medicago truncatula*)	8.00E–03	1.6	Defence	S	Extracellular	Sol
1844	gi|922403681	Chitinase / Hevein / PR-4 / Wheatwin2 (*Medicago truncatula*)	2.20E–02	2	Defence	S	Extracellular	Sol
2242	gi|922403685	Chitinase / Hevein / PR-4 / Wheatwin2 (*Medicago truncatula*)	5.10E–05	3	Defence	S	Extracellular	Sol
2252	gi|922403685	Chitinase / Hevein / PR-4 / Wheatwin2 (*Medicago truncatula*)	2.02E–04	3.3	Defence	S	Extracellular	Sol
1666	gi|922338023	pathogenesis-related thaumatin family protein (*Medicago truncatula*)	4.00E–03	5	defence	S	Extracellular	Sol
1516	gi|922390231	auxin-binding protein ABP19a (*Medicago truncatula*)	3.00E–02	1.5	nutrient reserve	S	Extracellular	Sol
1306	gi|357511665	rhicadhesin receptor (*Medicago truncatula*)	8.53E–04	2.2	nutrient reserve	S	Extracellular	Sol

843	gi|537317	peroxidase (*Medicago sativa*)	4.10E–02	1.5	oxidation-reduction process	S	Extracellular	Sol
848	gi|537317	peroxidase (*Medicago sativa*)	9.25E–04	1.8	oxidation-reduction process	S	Extracellular	Sol
849	gi|537317	peroxidase (*Medicago sativa*)	1.94E–04	2.6	oxidation-reduction process	S	Extracellular	Sol
854	gi|537317	peroxidase (*Medicago sativa*)	2.00E–03	2.1	oxidation-reduction process	S	Extracellular	Sol
2240	gi|537317	peroxidase (*Medicago sativa*)	5.93E–04	1.9	oxidation-reduction process	S	Extracellular	Sol
2241	gi|537317	peroxidase (*Medicago sativa*)	6.05E–04	1.9	oxidation-reduction process	S	Extracellular	Sol
2244	gi|537317	peroxidase (*Medicago sativa*)	8.00E–03	3.2	oxidation-reduction process	S	Extracellular	Sol
959	gi|357448431	peroxidase family protein (*Medicago truncatula*)	2.80E–02	1.2	oxidation-reduction process	S	Extracellular	Sol
900	gi|971560	peroxidase1B (*Medicago sativa*)	3.60E–02	1.4	oxidation-reduction process	S	Extracellular	Sol
958	gi|971560	peroxidase1B (*Medicago sativa*)	2.10E–02	1.6	oxidation-reduction process	S	Extracellular	Sol
2261	gi|971560	peroxidase1B (*Medicago sativa*)	4.00E–03	1.9	oxidation-reduction process	S	Extracellular	Sol
1250	gi|357437715	papain family cysteine protease (*Medicago truncatula*)	8.00E–03	1.7	proteolysis	S	Lysosome/Vacuole	Sol
